# Brain Activity During the Gradual‐Onset Continuous Performance Task in Adolescents: An Exploratory fMRI Study

**DOI:** 10.1002/brb3.71516

**Published:** 2026-06-04

**Authors:** Jade Awada, Natalia Fernandez, Vanessa Siffredi, Jenifer Miehlbradt, Cristina Borradori‐Tolsa, Maria Chiara Liverani, Russia Ha‐Vinh Leuchter

**Affiliations:** ^1^ Division of Development and Growth, Department of Paediatrics, Gynaecology and Obstetrics Geneva University Hospitals Geneva Switzerland; ^2^ Neuroimaging and Translational Psychiatry lab, Department of Psychiatry University of Geneva Geneva Switzerland; ^3^ Department of Radiology and Medical Informatics, Faculty of Medicine University of Geneva Geneva Switzerland

**Keywords:** adolescents, functional MRI, gradual‐onset continuous performance task, neuroimaging, sustained attention

## Abstract

**Introduction:**

Sustained attention (SA) and inhibition are fundamental processes that mature during adolescence, a developmental period between childhood and adulthood of rapid behavioral and cognitive changes. The current study aimed to provide a better understanding of SA and inhibition processes in typically developing adolescents (*n =* 26) aged 11‐18 years.

**Methods:**

Functional magnetic resonance images (fMRI) were acquired during two different modalities (*face* and *scene*) from a previously validated gradual‑onset continuous (gradCPT) paradigm to evaluate SA performance. In addition, we performed linear regression analyses to examine the relationship between covariates of interest and brain activation.

**Results:**

This exploratory study encountered performance challenges. Target detection accuracy was markedly lower than adults’ (*face*: 22.1%, *scene*: 28.1% vs. 74%–80% in adults), while non‐target accuracy remained relatively preserved (*face*: 86.5%, *scene*: 93.9%). Participants demonstrated better performance in the *scene* compared to the *face* condition (higher sensitivity d': t(25) = ‒5.11, *p* < 0.001; lower reaction time variability: t(25) = 4.99, *p* < 0.001). Despite poor behavioral performance, we observed activation in bilateral fronto‐parieto‐occipital regions during attempted response inhibition, regardless of modality. During the *face* gradCPT, activation showed a mainly left‐lateralized pattern in fronto‐cingulo‐cerebellar areas, while the *scene* gradCPT elicited more extensive bilateral fronto‐temporo‐parieto‐occipital activation. Brain activity correlated with behavioral performance measures (*p* < 0.002), though interpretation is constrained by low accuracy rates.

**Conclusion:**

Using the gradCPT paradigm, this exploratory study found activation in brain regions commonly linked to sustained attention and inhibition. However, these neural findings must be interpreted cautiously and cannot conclusively characterize sustained attention networks due to performance challenges. Our results highlight important methodological points for developmental applications and provide insights into the implementation of adult paradigms on younger audiences, such as adolescents.

## Graphical abstract

This exploratory fMRI study investigated neural mechanisms of sustained attention and inhibition in adolescents using two modalities of the gradCPT tasks. Brain activation during response inhibition showed modality‐specific patterns: face modality engaged left fronto‐cingulo‐cerebellar regions while scene modality activated bilateral fronto‐temporo‐parieto‐occipital networks.

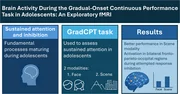

## 1 Introduction

Sustained attention (SA) is the ability to maintain stable and effective levels of attention over an extended period of time and is essential for many daily activities (Langner and Eickhoff 2012; Posner and Petersen 1990; Fortenbaugh et al. 2017). An attentional function closely related to SA is inhibition, defined as the ability to suppress impulsive responses in favor of appropriate ones (Carlson 2005; Reck and Hund 2011). Both sustained attention and inhibition processes begin to develop early in childhood and continue to mature throughout adolescence (Anderson et al. 2001; Best and Miller 2010; Fortenbaugh et al. 2015; Gallen et al. 2023; Leon‐Carrion et al. 2004; Luciana et al. 2005; Luna et al. 2004), a transitional period marked by rapid behavioral, socio‐emotional, and cognitive development.

Neural mechanisms of SA can be explored through functional magnetic resonance imaging (fMRI) coupled to a variety of tasks. One example of such tasks is the continuous performance task (CPT) (Riccio et al. 2001; Rosvold et al. 1956), where a rapid series of stimuli are presented at short intervals (∼1 s) (Rosenberg et al. 2013). In these tasks, participants have to respond only to rare target stimuli in a stream of frequent non‐target stimuli, which enables recording rare responses during short‐lasting phases of increased response latencies (Fortenbaugh et al. 2017; Rosenberg et al. 2013; Davies and Parasuraman 1982; Szalma et al. 2006; Warm and Jerison 1984; Fortenbaugh et al. 2018). Researchers have modified this design by changing the response pattern: participants now “respond to frequent non‐target stimuli and withhold responses to rare target stimuli” (Fortenbaugh et al. 2017; Rosenberg et al. 2013; Fortenbaugh et al. 2018; Conners 2000; Robertson et al. 1997). Building upon this approach, the gradual‐onset continuous performance task (gradCPT) was developed (Esterman et al. 2013). In the gradCPT, the switch between stimuli is gradual (i.e., a new image starts to emerge as the previous one fades), abrupt stimulus onsets are removed, and task demands are increased. This keeps participants engaged and allows for the investigation of reaction times (Fortenbaugh et al. 2017; Rosenberg et al. 2013; Esterman et al. 2013). Since its introduction, the gradCPT has been used in a variety of studies to assess SA in both typical (Rosenberg et al. 2013; Fortenbaugh et al. 2018; Esterman et al. 2014; Esterman et al. 2017; Kucyi et al. 2016) and clinical populations (Auerbach et al. 2014; Fortenbaugh et al. 2017; Rosenberg et al. 2016).

Key brain regions involved in SA have been reported in the literature of brain imaging studies of healthy adults (Langner and Eickhoff 2012; Fortenbaugh et al. 2017; Fisher 2019). They are mainly localized in the prefrontal cortex (i.e., medial, bilateral inferior, right midlateral, bilateral pre‐supplementary motor area (SMA), left dorsal premotor cortex, and bilateral ventral premotor cortex), the anterior insula, and the midcingulate cortex. Other areas were found in the temporal and parietal areas (i.e., right temporo‐parietal junction (TPJ), temporal lobule, and intraparietal sulcus), as well as in the occipital areas (i.e., right occipital gyri and left temporo‐occipital junction), the cerebellum, and subcortical structures (i.e., bilateral thalamus). Using CPTs and go/no‐go, a SA network similar to the one observed in adults was identified in healthy children and adolescents (Morandini et al. 2020). Despite the gradCPT task having been used to assess SA in adults (Esterman et al. 2013; Esterman et al. 2016; Jun and Lee 2021) and in populations of adolescents at risk (Auerbach et al. 2014), it has not yet been used to investigate the brain correlates of SA and inhibition in typically developing adolescents.

Therefore, given the limited research in typically developing adolescents, we conducted an exploratory investigation to examine the feasibility of adapting the adult‐validated fMRI gradCPT paradigm to this population, using two distinct modalities: *face* and *scene* (Rosenberg et al. 2016). Both modalities were included to investigate whether performance and neural patterns differed among stimulus categories that activate different attentional processing pathways. In this exploratory approach, we investigated their behavioral performance and brain activation patterns and examined the association between executive and attention skills and brain activation during SA. This exploratory approach aimed to assess both the applicability of the gradCPT in this age group and identify potential methodological considerations for developmental applications of this paradigm.

## 2 Methods

A full description of the methods, including data collection, exclusion criteria, detailed examination of executive and attentional assessment, neuroimaging protocol and analyses, as well as statistical analysis, can be found in S1 of supplemental materials (SM).

### 2.1 Participants

Twenty‐nine adolescents from 11 to 18 years old (M = 15 years, SD = 2) were recruited from the community between January 2021 and August 2022. All adolescents were typically developing and attended standard schools. Participants with an intelligence quotient below 70, any sensory or physical impairments (e.g., blindness, hearing loss, cerebral palsy), or an insufficient understanding of French or English were excluded. Written informed consent was obtained from all participants and their principal caregiver in accordance with regulation of the ethics committee at the University Hospital of Geneva and the Swiss Ethics Committee (ID: 2015‐00175). General intellectual functioning was derived from the General Ability Index (GAI) of the Wechsler Intelligence Scale for Children ‐ fifth Edition (WISC‐V) (Wechsler 2014). Socio‐economic status was estimated using the Largo score (Largo et al. 1989) (see S1 in SM for details on both scales).

### 2.2 Executive and Attentional Behavioral Measures

Adolescents’ executive and attentional abilities were assessed using three standardized tools: (1) The Conners’ Rating Scale, third edition (Conners 3‐SR) self‐report questionnaire; (2) the parent‐completed Behavior Rating Inventory of Executive Functioning (BRIEF) questionnaire; (3) selected subtests from the Test of Attentional Performance (TAP).

For the BRIEF questionnaire, we used the Behavioral Regulation Index (BRI), the Metacognition Index (MI), and the Global Executive Composite index (GEC). In addition, the TAP included two Go/No‐Go paradigms. Full descriptions of each measure are provided in S1 in SM.

### 2.3 Experimental fMRI Paradigm: Gradual‐Onset Continuous Performance Task

**FIGURE 1:**
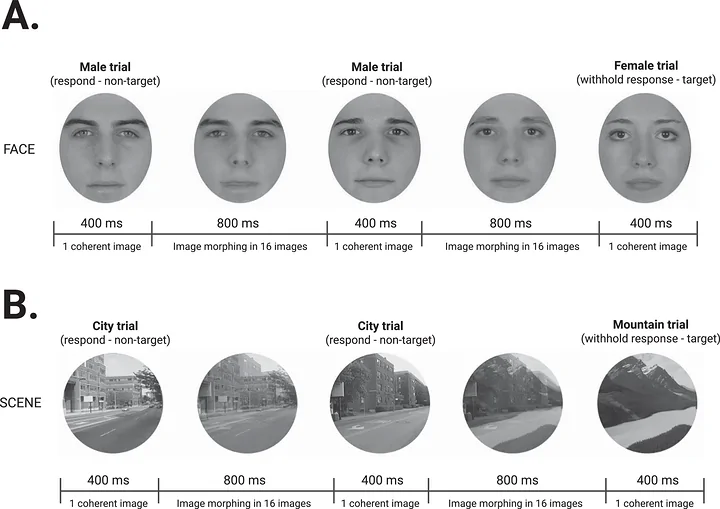
GradCPT. Illustration of the gradCPT paradigm for both (A) face and (B) scene modalities. Target trials (female faces and mountains) are presented 10% of the time and must be withheld, while non‐target trials (male faces and city scenes) are presented 90% of the time and must be answered by pressing a button. In our fMRI implementation, each trial had an effective duration of approximately 600 ms (500 task volumes × TR 720 ms / 600 trials), with stimuli gradually transitioning from one trial to the next. Note that the 1,200 ms trial structure (800 ms transition + 400 ms pause at full coherence) illustrated here reflects the original Rosenberg et al. (2013) protocol and served as the basis for our paradigm design (Fernandez et al. 2024, in prep, Rosenberg et al. 2013).

The experimental paradigm consists of two distinct versions of the gradCPT: the *face* and *scene* modalities (Rosenberg et al. 2013; Rosenberg et al. 2016). In each modality, stimuli gradually transitioned between the 60 target (T) (e.g., female faces and mountains) and 540 non‐target (NT) (e.g., male faces and cities) trials at a constant rate (see Figure 1). Participants responded as fast and as accurately as possible to NT stimuli by pressing the response button and withheld responses to T stimuli (Rosenberg et al. 2013; Fernandez et al. 2024). All participants performed the task in the scanner with the right hand. The order of the two modalities was counterbalanced across participants (*n =* 13 completed the *face* modality first; *n =* 13 completed the *scene* modality first). Further details about the task, including stimulus timing and trial structure, are provided in the SM.

### 2.4 Behavioral Data Analyses

SA performance was assessed by the percentage of correct answers (*accuracy*, AC), sensitivity scores (*d’*), and the reaction time coefficient of variation (RT_CV_). All statistical analyses were performed using RStudio version 4.2.1 (R Core Team 2022) and Statistica software version 14.0.0 (TIBCO 2020).

For each participant and modality, we computed the AC for both NT and T trials, which were analyzed using a Mann‐Whitney test with modality (*face* and *scene*) and stimuli (NT and T) being the within‐subject levels.

*d’* scores were calculated individually for each participant and modality (Rosenberg et al. 2013; Rosenberg et al. 2016; Rosenberg et al. 2015) (see S1 in SM for calculation details). A simple t‐test on d’ scores was then performed to compare SA performance across modalities.

To assess response consistency, the RT_CV_ for correct NT stimuli was computed independently for each participant within each modality. Differences in RT_CV_ between modalities were analyzed using simple t‐tests.

Furthermore, we examined the influence of age, socio‐economic status (measured by the Largo scale), executive functions (evaluated with the BRIEF), and attentional abilities (evaluated with the Conners 3‐SR and TAP) on SA performance. To examine the influence of covariates on SA performance while accounting for the within‐subject repeated measures structure (i.e., *face* and *scene* modalities per participant), we conducted a linear mixed‐effects model (fitlme, MATLAB Statistics Toolbox, version R2022a) with RTCV as the dependent variable, Task (*face* vs. *scene*), age, socio‐economic status, IAG, BRIEF subscales (GEC, MI, BRI), Conners 3‐SR (inattention and hyperactivity), and TAP scores (divided attention, Go/No‐Go simple and hard, and working memory) as fixed effects, and Subject as a random intercept. A separate analysis with d' as the dependent variable was also conducted. *P*‐values were corrected for multiple comparisons using the Benjamini‐Hochberg FDR procedure.

### 2.5 FMRI Analyses

#### 2.5.1 MRI Acquisition Parameters

MRI data were acquired at the Campus Biotech (Geneva, Switzerland) using a Siemens 3T Magnetom Prisma scanner and a standard 64‐channel head coil. The parameters for acquisition included a T1‐weighted magnetization‐prepared rapid gradient‐echo (MP‐RAGE) with a voxel size of 0.9 × 0.9 × 0.9 mm and a repetition time/echo time (TR/TE) of 2300/ 2.32 ms, as well as a T2*‐weighted images using multi‐slice gradient‐echo‐planar imaging (EPI) sequences with a voxel size of 2 × 2 × 2 mm, and a TR/TE of 720/33 ms. Further acquisition details are provided in S1 in SM.

#### 2.5.2 Preprocessing

fMRI images were preprocessed using an in‐house preprocessing pipeline (Freitas et al. 2021; Liverani et al. 2020), which included steps for realignment and unwarping (Hutton et al. 2002), co‐registration, normalization to a DARTEL template (Ashburner 2007), and smoothing with a 6‐mm full width at half maximum (FWHM) Gaussian kernel. Temporal high‐pass filtering was applied using SPM's default filter with a cutoff of 128 s to remove low‐frequency drifts. Head motion was assessed with Framewise Displacement (FD) (Power et al. 2012), calculated for each modality separately and used as an exclusion criterion (see S1 in SM for more details).

#### 2.5.3 FMRI Analysis

Individual first‐level analyses were performed with a General Linear Model (GLM), incorporating task conditions (T and NT) in each modality. Individual t‐contrasts were computed by comparing the two experimental conditions (T > NT) and modalities (*face* > *scene* or *scene* > *face*) (Fernandez et al. 2024).

Second‐level (whole‐brain) analyses were conducted using a flexible factorial model comprising modalities (*face* and *scene*), stimuli (T and NT), and subject as separate factors. We focused on: 1) the main effect of response inhibition (T > NT) and 2) its modulation by task modality (*face* (T > NT) > *scene* (T > NT) or *scene* (T > NT) > *face* (T > NT)).

The direction of the interaction was confirmed by applying the effect of response inhibition for a specific task (*face* (T > NT) or *scene* (T > NT), respectively) as an inclusive mask (Fernandez et al. 2024). Moreover, we examined univariate linear regression to determine the association between cerebral activity during response inhibition and covariates of interest (i.e., *d’* scores, RT_CV_, age at testing, and socioeconomic status, as well as BRIEF, Conners 3‐SR, and TAP scores). Individual beta values, extracted from these analyses using spheres (27 voxels) centered on the main peak coordinates, were used for further analyses.

*P*‐values were thresholded at p_unc _< 0.001 with a minimum cluster size of 50 voxels, thus uncorrected for multiple comparisons (Lieberman and Cunningham 2009). All findings are presented in MNI space.

## 3 Results

### 3.1 Participants’ Characteristics

After application of exclusion criteria (see S1 in SM for the detailed explanation), 26 adolescents were included for the final analysis. The sample was sex‐balanced (13 males, 13 females), had a mean General Ability Index of 110.36 (SD = 11.11) and a mean socio‐economic status of 3.00 (SD = 1.57). Overall, neurobehavioral scores of executive and attention functions ranged in the average range (see Table 1).

**TABLE 1:** Executive and attentional behavioral results (A) and GradCPT behavioral performances for the Face and the Scene tasks (B).

**A) Demographic data**					
	**Males (*N =* 13)**		**Females (*N =* 13)**		**Group comparison**
	Mean	SD	Mean	SD	t and p_val_
Age	14.51	1.8	15.53	2.2	*t =* ‒1.292, *p =* 0.209
Right‐handedness, N (%)	12 (92.3%)		12 (92.3%)		
**B) Executive and attentional neurobehavioral results**					
Parent‐reported BRIEF questionnaire					
	Mean T scores	SD	Mean T scores	SD	t and p_val_
Global executive composite index (GEC)	54.62	9.7	49.77	11.9	*t =* 1.143, *p =* 0.264
Metacognition Index (MI)	53.77	8.8	50.77	12.9	*t =* 0.693, *p =* 0.495
Behavioral regulation index (BRI)	54.62	13.6	47.54	9.9	*t =* 1.521, *p =* 0.141
*Conners 3‐SR*					
Inattention	57.46	8.2	52.15	11.7	*t =* 1.337, *p =* 0.194
Hyperactivity	57.92	11.1	55.62	12.5	*t =* 0.498, *p =* 0.623
*Neuropsychological testing—TAP*					
Go/No‐Go (2 stimuli, 1 target) (Nb of errors)	43.85	6.1	41	8.0	*t =* 1.023, *p =* 0.317
Go/No‐Go (5 stimuli, 2 targets) (Nb of errors)	46.23	8.1	47.31	7.0	*t =* −0.362, *p =* 0.721

**C) GradCPT behavioral performances**	**Face task**	**Scene task**	**Task comparison**
**(a) Accuracy**			
Percentage of Correct Non‐Target	86.5% (14%)	93.9 (7%)	W = 246.5, *p =* 0.096
Percentage of Correct Target	22.1% (17%)	28.1 (22%)	W = 288.5, *p =* 0.369
**(b) Sensitivity scores (d’)**			
d’ = z(HIT rate)—z(FALSE ALARM rate)	0.44 (0.43)	1.12 (0.59)	t(25) = ‒5.11, *p* < 0.001
**(c) Reaction time variability (RT_CV_)**			
RT_CV_ = standard deviation of RT/mean RT	0.29 (0.04)	0.25 (0.04)	t(25) = 4.99, p < 0.001

*Note*: A) Demographic data: age of participants across groups (i.e., males and females). B) Executive and attentional scores result from the parent‐reported BRIEF questionnaire, the self‐reported Conners 3‐SR, and the TAP are reported in T scores. C) Mean accuracy (a), mean sensitivity scores (b), and reaction time variability (c) are presented with standard deviation in brackets. Statistical comparisons between the two tasks were performed using the Wilcoxon signed ‐rank test (W) for accuracy values and a paired‐sample Student t‐test (t) for sensitivity scores and reaction time variability.

### 3.2 Behavioral Results

Summary statistics of the behavioral results in gradCPT, including AC, d’, and RT_CV_, are shown in Table 1. Participants performed significantly better on the *scene* (i.e., higher *d’* scores, *p* < 0.001; lower RT_CV_, *p* < 0.001) compared to the *face* modality. AC rates were similar between the two modalities, both for NT (W  =  246.5, *p*  =  0.096) and T stimuli (W  =  288.5, *p*  =  0.369). However, AC was significantly higher for NT‐stimuli than for T‐stimuli (*p* < 0.001).

Additionally, the multiple linear regression of *d’* scores did not reveal a significant effect of any covariate of interest. In contrast, for RT_CV_ scores, a linear mixed effects model (LME; *N =* 52; random intercept per participant) revealed one significant predictor after FDR correction: the number of errors committed in the Go/No‐Go TAP task (2 stimuli, 1 target) [β = −0.003, t(38) = −3.37, *p* = 0.002, p (FDR) = 0.015]. Conners 3‐SR inattention scores showed a trend‐level association [β = 0.002, t(38) = 2.21, *p* = 0.036, *p* (FDR) = 0.157]. Task modality (*scene* vs. *face*) was also a significant fixed effect [β = −0.041, t(38) = −5.09, *p* (FDR) < 0.001], confirming better response consistency in the *scene* modality (see Table 2).

**TABLE 2:** Analyses of GradCPT behavioral performances, including covariates.

**Model: F(13,38) — AIC = −178.76; ICC = 0.178 (random intercept per subject)**						
**Predictor**	***β***	**SE(β)**	***t***	**df**	***p***	**p (FDR)**
Task (scene vs. face) *	−0.041	0.008	−5.086	38	< 0.001	**< 0.001**
Age	−0.0004	0.0003	−1.208	38	0.234	0.401
Largo (SES)	0.008	0.004	1.762	38	0.090	0.234
IAG	0.001	0.0004	2.061	38	0.049	0.161
GEC (BRIEF)	0.012	0.013	0.905	38	0.371	0.477
MI (BRIEF)	−0.009	0.009	−0.974	38	0.336	0.477
BRI (BRIEF)	−0.004	0.005	−0.724	38	0.473	0.515
Inattention (Conners)	0.002	0.001	2.208	38	0.036	0.157
Hyperactivity (Conners)	−0.001	0.001	−1.325	38	0.193	0.401
Divided Attention (TAP)	0.0004	0.0005	0.849	38	0.401	0.477
Go/No‐Go simple (TAP) *	−0.003	0.001	−3.374	38	0.002	**0.015**
Go/No‐Go hard (TAP)	0.001	0.001	1.186	38	0.243	0.401
Working Memory (TAP)	−0.0002	0.001	−0.271	38	0.788	0.789

*Note*: Linear mixed‐effects model (random intercept per subject) predicting RTcv. All covariates and task (*face* vs. *scene*) were entered simultaneously as fixed effects (*N =* 52; 26 participants × 2 modalities).

Mixed‐effects model with random intercept per subject (fitlme, MATLAB).

Rows marked with * are significant after FDR correction (*p* < 0.05).

Abbreviations: β = standardized estimate; β BRIEF = Behavior Rating Inventory of Executive Function (BRI = Behavior Regulation Index, GEC = Global Executive Composite, MI = Metacognition Index); FDR = Benjamini‐Hochberg correction applied to all fixed effects (excluding intercept); IAG = General Ability Index; TAP = Test of Attentional Performance.

*p(FDR) < 0.05.

### 3.3 Whole‐Brain Activations During the gradCPT Paradigm

**FIGURE 2:**
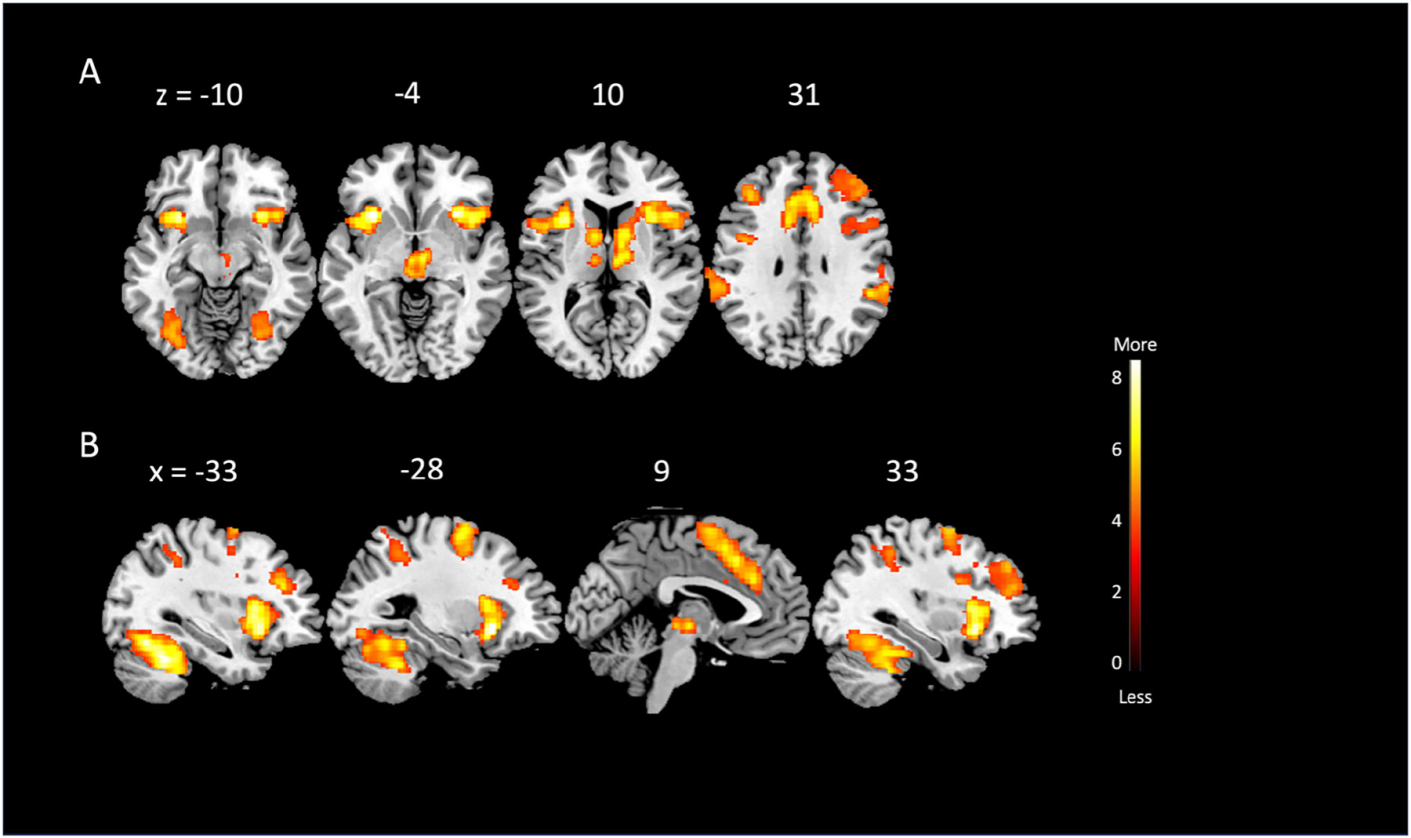
Cerebral areas engaged during response inhibition trials (Target > Non‐Target). All clusters are significant at peak level at *p* < 0.001, uncorrected for multiple comparisons, with a minimum size of 50 voxels. Activations are displayed on a template image and numbers indicate z (A. axial view) and x (B. sagittal view) coordinates in an MNI space.

The main effect of response inhibition, regardless of the type of modality, revealed a bilateral fronto‐parieto‐occipital pattern of brain activity (see Table 3, Figure 2). This pattern included frontal regions (the frontal operculum, the inferior, superior, and middle frontal gyrus (IFG, SFG, and MFG, respectively), the precentral gyrus, and motor areas); parietal regions (both the inferior and superior parietal lobule (the IPL and the SPL, respectively) and the supramarginal gyrus (SMG); occipital regions (the fusiform gyrus and the middle occipital gyrus (MOG)); as well as the anterior cingulate cortex (ACC) and the midcingulate cortex (MCC), the anterior insula, the cerebellum, and the thalamus.

**TABLE 3:** Regional cerebral activations during response inhibition regardless of the type of task (Target > Non‐Target).

Cerebral regions			MNI coordinates				
		Lat.	x	y	z	Cluster size	Z_E_
**Target ≥ Non‐Target**							
Frontal	Frontal operculum	L	‒32	16	10	1399	5.827*
		R	42	16	2	4457	5.170*
	Inferior frontal gyrus (*pars opercularis*)	R	50	18	0	4457	5.351*
	Inferior frontal gyrus (*pars triangularis*)	L	‒38	34	24	449	5.492*
	Middle frontal gyrus	L	‒32	26	28	449	4.319*
	Middle frontal gyrus	R	38	44	22	4457	4.733*
	Posterior‐medial frontal—dACC/SMA	R	14	6	70	5968	6.447*
	Inferior precentral gyrus	L	‒40	‒2	32	138	4.324*
	Inferior precentral gyrus	R	48	6	40	4457	4.539*
	Pre‐supplementary motor area	R	2	12	50	5968	5.299*
	Superior frontal gyrus	L	‒22	0	52	5968	6.075*
	Superior frontal gyrus	R	20	‒2	66	5968	5.681*
Subcortical	Basal ganglia—Caudate nucleus	L	‒8	2	8	276	5.340*
	Basal ganglia—Caudate nucleus	R	14	4	12	4457	5.506*
Parietal	Inferior parietal lobule	L	‒54	‒36	46	1247	4.616*
	Inferior parietal lobule	R	48	‒38	52	1759	5.353*
	Posterior intraparietal sulcus	R	28	‒64	48	1759	3.961
	Superior precuneus	L	‒8	‒66	54	183	3.959
	Superior precuneus	R	10	‒60	56	325	4.652*
	Superior parietal lobule	L	‒30	‒52	50	1247	4.065
	Superior parietal lobule	R	12	‒56	60	325	4.643*
	Inferior supramarginal gyrus	L	‒60	‒36	32	1247	4.460*
	Inferior supramarginal gyrus	R	50	‒40	32	1759	5.050*
Occipital	Inferior fusiform gyrus (area FG3)	L	‒30	‒48	‒18	1959	5.614*
	Inferior fusiform gyrus—FFA	L	‒34	‒64	‒16	1959	6.092*
	Inferior fusiform gyrus—FFA	R	36	‒48	‒22	1197	5.377*
	(Superior) fusiform gyrus—OFA	R	28	‒70	‒8	1197	4.299*
	Middle occipital gyrus	L	‒24	‒80	20	2706	5.497*
Other	Anterior cingulate gyrus	L	‒6	28	30	5968	5.179*
	Anterior cingulate gyrus	R	8	24	32	5968	5.289*
	Anterior insula	L	‒30	16	‒6	1399	7.223*
	Anterior insula	R	32	18	‒6	4457	6.542*
	Cerebellum (IV)	L	‒36	‒52	‒28	1959	6.632*
	Cerebellum (IV)	R	34	‒44	‒34	1197	5.004*
	Cerebellum (crus 1)	R	40	‒44	‒34	1197	5.427*
	Medial—anterior midcingulate cortex	L	‒8	14	40	5968	5.597*
	Medial—anterior midcingulate cortex	R	6	18	42	5968	5.754*
	Thalamus	L	‒8	‒16	8	81	4.594*
	Thalamus	R	8	‒16	10	4457	5.277*

*Note*: All reported clusters are significant at peak‐level at *p* < 0.001, uncorrected for multiple comparisons, with a minimum size of 50 voxels. Peaks that are significant after correction for multiple comparison are indicated with an asterisk (*FDR‐corrected).

Abbreviation: Lat.: Hemisphere lateralization (L = left; R = right) Z‐score (Z_E_) values refer to the activation maxima to the SPM coordinates.

**FIGURE 3:**
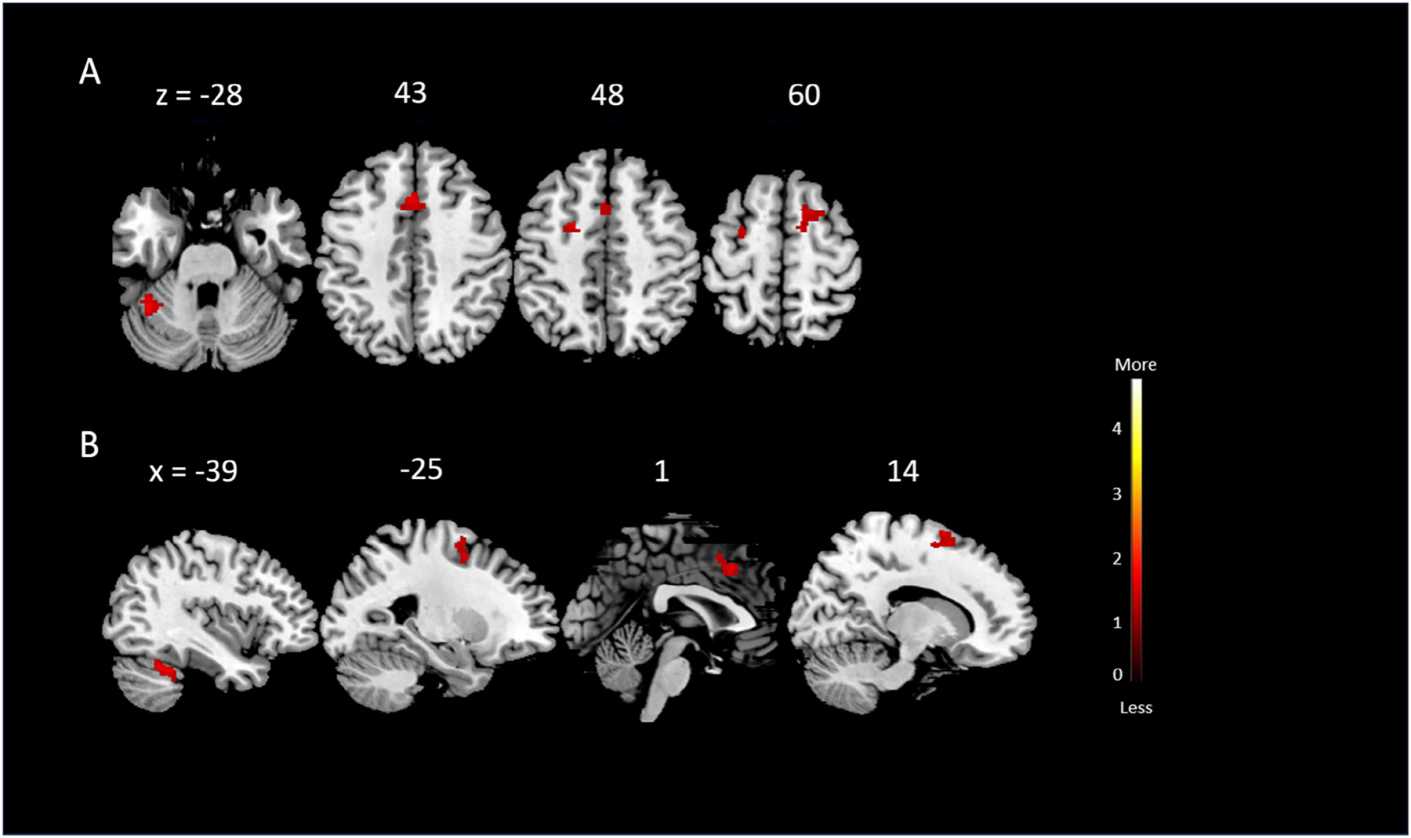
Cerebral areas engaged during response inhibition trials in the Face task (Target > Non‐Target). All clusters are significant at peak level at *p* < 0.001, uncorrected for multiple comparisons, with a minimum size of 50 voxels. Activations are displayed on a template image, and numbers indicate z (A. axial view) and x (B. sagittal view) coordinates in an MNI space.

**FIGURE 4:**
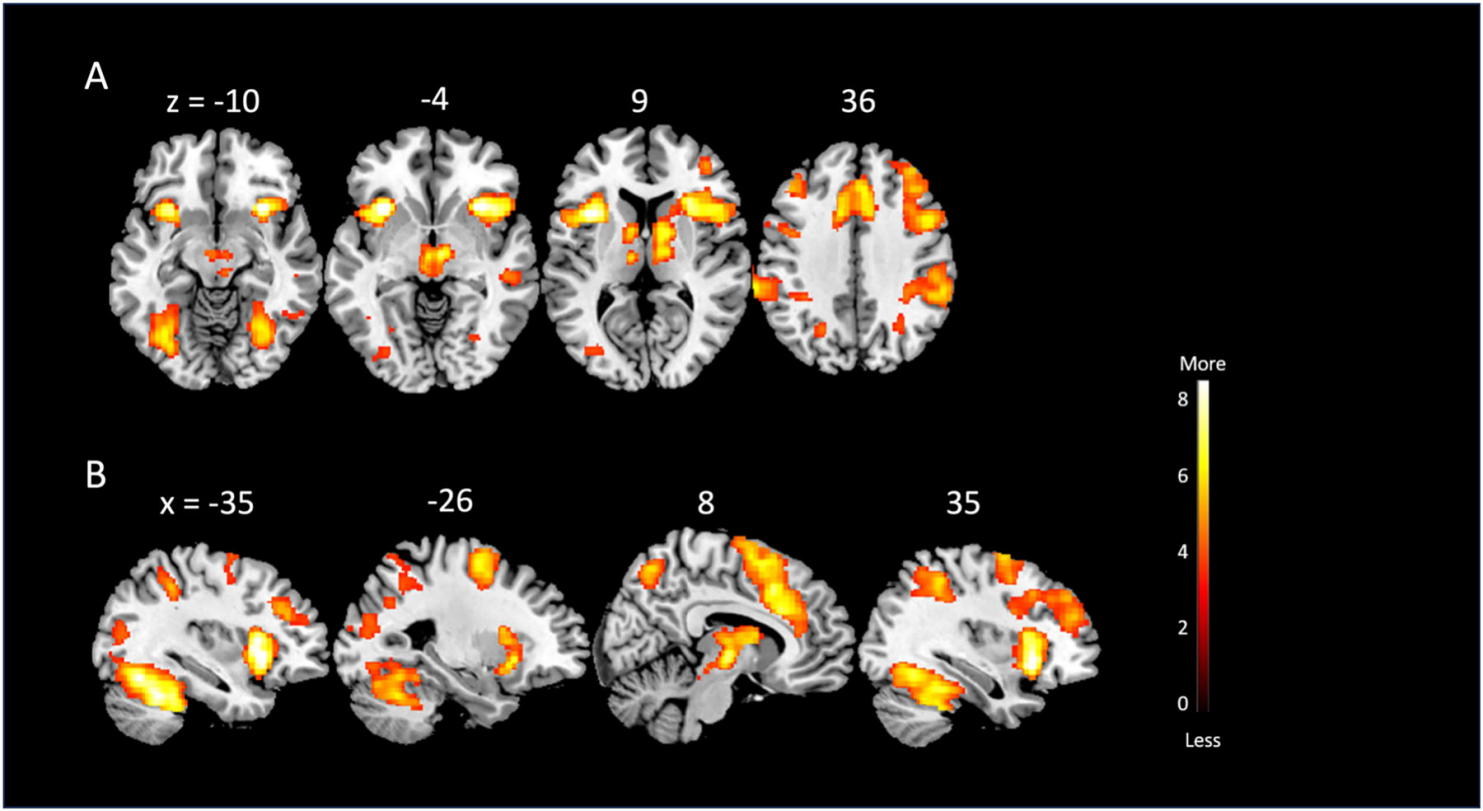
Cerebral areas engaged during response inhibition trials in the Scene task (Target > Non‐Target). All clusters are significant at peak level at *p* < 0.001, uncorrected for multiple comparisons, with a minimum size of 50 voxels. Activations are displayed on a template image, and numbers indicate z (A. axial view) and x (B. sagittal view) coordinates in an MNI space.

When considering each modality separately, the *face* gradCPT showed a mainly left‐lateralized activation pattern covering mainly frontal regions (the precentral gyrus, the SMA, and the SFG), the MCC, as well as the posterior cerebellum (see Table 4, Figure 3). In contrast, the *scene* gradCPT revealed a wider and bilateral pattern of brain activity across frontal (frontal gyri (inferior, middle, and superior), motor area, and precentral gyrus), temporal (the inferior and middle temporal gyri (ITG and MTG, respectively)), parietal (the intraparietal sulcus, both the IPL and SPL, precuneus, and the SMG), and occipital areas (inferior occipital gyrus (IOG), the MOG, and fusiform gyrus). It additionally revealed activity in the anterior cingulate cortex, the anterior insula, the cerebellum, and the thalamus (see Table 4 and Figure 4).

**TABLE 4:** Regional cerebral activations associated with performance of a response inhibition during the face and scene tasks.

	Cerebral regions		MNI coordinates				
			x	y	z	Cluster size	Z_E_
**Face (Target≥Non‐Target)**							
Frontal	Superior—precentral gyrus	L	‒26	‒2	60	118	3.394
	Posterior‐medial frontal/SMA	L	0	18	44	155	3.995
	Superior frontal gyrus	L	‒22	0	52	118	4.267
	Superior frontal gyrus	R	16	10	62	183	3.761
Other	Cerebellum (VI)	L	‒40	‒46	‒30	112	4.254
	Medial—anterior midcingulate gyrus	L	‒8	14	38	155	3.225
Scene (Target≥Non‐Target)							
Frontal	Inferior frontal gyrus	L	‒52	10	12	1499	4.695*
	(*p. Opercularis*)	R	50	18	0	12784	5.886*
	Middle frontal gyrus	L	‒40	36	26	524	5.230*
	Superior frontal gyrus	L	‒20	‒2	62	12784	5.733*
	Superior frontal gyrus	R	18	0	60	12784	5.725*
	Superior—precentral gyrus	L	‒60	8	30	1499	3.639
	Posterior‐medial—SMA	R	14	4	70	12794	6.414*
	Medial—pre‐SMA	L	‒10	12	42	12784	5.823*
Temporal	Inferior temporal gyrus	R	50	‒56	‒14	1796	3.947
	Middle temporal gyrus	R	50	‒28	‒4	96	4.083
Parietal	Anterior—intraparietal sulcus	L	‒24	‒58	46	1906	3.761
	Inferior parietal lobule	L	‒40	‒44	46	1906	4.908*
	Inferior parietal lobule	R	48	‒38	52	2875	6.337*
	Superior parietal lobule	L	‒30	‒50	48	1906	4.144
	Superior parietal lobule	R	28	‒64	48	2875	4.768*
	Superior—precuneus	R	10	‒64	56	381	5.058*
	Superior—supramarginal gyrus	L	‒62	‒48	42	1906	3.672
	Superior—supramarginal gyrus	R	62	‒38	30	2875	5.363*
Occipital	Inferior occipital gyrus	L	‒33	‒84	0	2863	3.363
	Middle occipital gyrus	L	‒30	‒78	22	2863	4.219*
	Superior—fusiform gyrus	L	‒30	‒48	‒18	2863	6.070*
	Inferior—fusiform gyrus (FFA)	L	‒34	‒64	‒16	2863	6.610*
	Inferior—fusiform gyrus (FFA)	R	34	‒62	‒14	1796	5.613*
Other	Medial—anterior cingulate cortex	R	8	24	28	12794	5.790*
	Lateral—anterior cingulate cortex	R	8	18	42	12794	5.933*
	Anterior insula	L	‒36	12	2	1499	7.235*
	Anterior insula	R	32	18	‒8	12794	7.586*
	Cerebellum (VI)	L	‒32	‒54	‒32	2863	6.138*
	Cerebellum (VI)	R	34	‒54	‒30	1796	5.264*
	Cerebellum (crus 1)	L	‒40	‒56	‒28	2863	6.748*
	Cerebellum (crus 1)	R	40	‒44	‒34	1796	5.682*
	Thalamus	R	6	‒12	‒2	12794	5.985*

*Note*: Clusters are significant at peak level at *p* < 0.001, uncorrected for multiple comparisons, with a minimum size of 50 voxels. Clusters that are significant after correction for multiple comparison are indicated with an asterisk (*FDR‐corrected).

Abbreviation: Lat.: Hemisphere lateralization (L = left; R = right) Z‐score (Z_E_) values refer to the activation maxima to the SPM coordinates.

### 3.4 Associations Between Brain Activation Related to Response Inhibition and Behavioral Measures

Multiple linear regressions with covariates were performed for each modality separately to assess the effect of individual differences in demographic characteristics (i.e., age at testing and socio‐economic status) and executive and attentional abilities (*d’*, RT_CV_, well as BRIEF, Conners 3‐SR scores, and TAP scores) on response inhibition brain activity.

In the *face* gradCPT, we found positive associations between RT_CV_ (i.e., higher reaction time variability) and increased activity in frontal (bilateral superior frontal gyrus and dACC/SMA), parietal (bilateral angular gyrus), and temporal regions (left MTG and the left STG) (see Table 5 and Table SM1 in SM). However, we did not find any association between *d’* scores and response inhibition‐induced brain activity. There were also positive associations between Conners’ inattention scores and brain activation in frontal (the superior precentral gyrus), temporal (the STG), parietal (the superior precuneus and the SPL), occipital (the calcarine sulcus, the superior cuneus, the lingual gyrus, the superior occipital gyrus (SOG)), and insula lobes (see Table 6 and Table SM2 in SM). Additionally, we found positive correlations with the number of errors in the Go/No‐Go task (2 stimuli – 1 target) and brain activity in parietal (the inferior precuneus), occipital (the inferior cuneus), and subcortical brain regions (the putamen). One positive correlation with the number of errors in the Go/No‐Go task (5 stimuli – 2 targets) was found in the superior precentral gyrus (see Table 7 and Table SM3 in SM).

**TABLE 5:** Regional cerebral activations associated with behavioral measures (d’ and RT_CV_) during the Face and Scene task during response inhibition (Target>Non‐Target).

	Cerebral regions		MNI coordinates				
		Lat.	x	y	z	Cluster size	Z_E_
**Face task**							
**Positive correlation with RT_CV_**							
Frontal	Superior frontal gyrus	L	‒2	36	34	86	3.186
	Superior frontal gyrus	R	8	32	42	86	4.044
	Posterior—medial frontal—dACC/SMA	R	8	30	52	86	3.273
Temporal	Middle temporal gyrus	L	‒56	‒46	6	91	3.754
	Superior temporal gyrus	L	‒50	‒38	6	91	4.095
Parietal	Angular gyrus	L	0	36	20	49	4.022
	Angular gyrus	R	42	‒62	54	304	4.150
							
**Scene task**							
**Positive correlation with d’**							
Other	Anterior cingulate cortex	L	0	36	20	49	4.022
**Positive correlation with RT_CV_**							
Other	Cerebellum (VI)	L	‒16	‒66	‒20	51	4.199

*Note*: Clusters are significant at peak level at *p* < 0.001, uncorrected for multiple comparisons, with a minimum size of 50 voxels.

Abbreviation: Lat.: Hemisphere lateralization (L = left), Z‐score (Z_E_) values refer to the activation maxima to the SPM coordinates.

**TABLE 6:** Regional cerebral activations associated with Conners’ inattention scores during the Face and Scene tasks during response inhibition (Target>Non‐Target).

Cerebral regions			MNI coordinates				
		Lat.	x	y	z	Cluster size	Z_E_
**Face task**							
**Positive correlation with Conners’ inattention scores**							
Frontal	Superior precentral gyrus	L	‒4	‒22	68	113	4.004
Temporal	Superior temporal gyrus	L	‒54	‒20	‒8	139	4.292
Parietal	Superior precuneus	L	‒12	‒50	68	54	4.826
	Superior parietal lobule	L	‒22	‒64	34	53	3.524
	Superior parietal lobule	R	24	‒52	58	68	4.199
Occipital	Calcarine sulcus	L	‒22	‒70	8	107	4.097
	Calcarine sulcus	R	14	‒74	12	204	4.287
	Superior cuneus	L	‒6	‒86	40	100	3.712
	Superior cuneus	R	14	‒78	22	204	3.659
	Lingual gyrus	L	‒24	‒56	‒2	107	4.097
	Superior occipital gyrus	L	‒20	‒84	34	100	4.107
	Superior occipital gyrus	R	24	‒76	36	101	4.113
Other	Insula lobe—posterior insula	R	36	‒16	20	61	4.050
							
**Scene task**							
**Positive correlation with Conners’ inattention scores**							
Parietal	Precuneus	L	2	‒76	40	128	4.223
Occipital	Calcarine sulcus	L	‒2	‒78	6	791	3.961
	Inferior cuneus	L	0	‒74	10	791	3.921
	Inferior cuneus	R	8	‒92	10	791	4.643
	Inferior lingual gyrus	L	‒14	‒68	‒2	185	4.182
	Inferior lingual gyrus	R	8	‒60	0	128	4.056
	Superior occipital gyrus	L	‒8	‒86	42	128	5.179
	Superior occipital gyrus	R	12	‒90	32	791	3.625
Other	Cerebellum (VI)	L	‒8	‒64	‒8	185	4.042

Clusters are significant at peak level at p < 0.001, uncorrected for multiple comparisons, with a minimum size of 50 voxels.

Abbreviation: Lat.: Hemisphere lateralization (L = left), Z‐score (Z_E_) values refer to the activation maxima to the SPM coordinates.

**TABLE 7:** Regional cerebral activations associated with the number of errors in the Go/No‐Go task of the TAP during the Face and Scene tasks during response inhibition (Target>Non‐Target).

Cerebral regions			MNI coordinates				
		Lat.	x	y	z	Cluster size	Z_E_
**Face task**							
**Positive correlation with the number of errors in Go/No‐Go (2 stim – 1 target)**							
Parietal	Inferior precuneus	L	‒20	‒62	22	114	3.803
Occipital	Inferior cuneus	L	‒16	‒66	18	114	3.839
Subcortical	Basal ganglia—putamen	L	‒26	‒12	10	53	4.350
**Positive correlation with the number of errors in Go/No‐Go (5 stim – 2 targets)**							
Frontal	Superior precentral gyrus	L	‒32	‒18	64	54	3.985
							
Scene task							
**Positive correlation with the number of errors in Go/No‐Go (5 stim – 2 targets)**							
Temporal	Superior temporal gyrus	R	54	‒16	4	117	4.497
Other	Insula lobe—posterior insula	R	38	‒8	4	56	3.781

There are two types of Go/No‐Go task: one with 2 presented stimuli and 1 target (Go/No‐Go (2 stim – 1 target)) and one with 5 presented stimuli and 2 targets (Go/No‐Go (5 stim – 2 targets)). Clusters are significant at peak‐level at p < 0.001, uncorrected for multiple comparisons, with a minimum size of 50 voxels.

Abbreviation: Lat.: Hemisphere lateralization (L = left), Z‐score (Z_E_) values refer to the activation maxima to the SPM coordinates.

**FIGURE 5:**
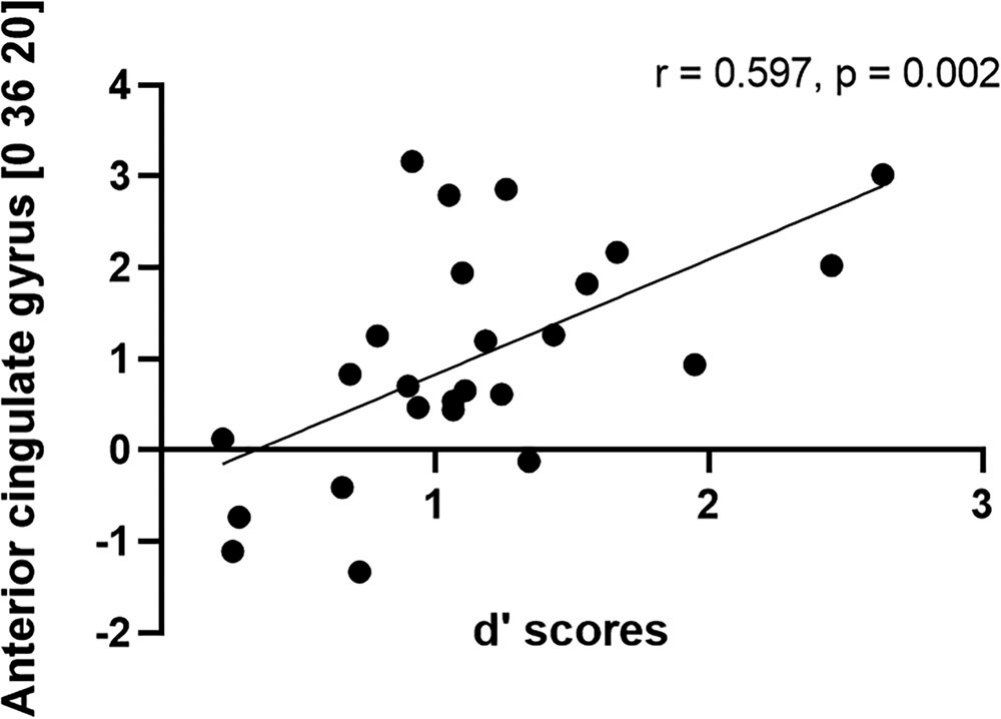
Association of task‐induced activation in the anterior cingulate gyrus with (d’) scores.

Regarding the *scene*, gradCPT, we found additional positive associations between behavioral performance and brain activation. Specifically, participants with higher *d’* scores activated more the anterior cingulate, while participants with higher RT_CV_ exhibited larger brain activation in the left posterior cerebellum (see Table 5, Figure 5, and Table SM1 in SM). Concerning attentional abilities, a correlation was found between Conners’ inattention scores and task‐related brain activation in parietal regions (precuneus), occipital regions (the calcarine sulcus, the inferior cuneus, the inferior lingual gyrus, and the SOG), and the posterior cerebellum (see Table 6 and Table SM2 in SM). Finally, the number of errors in the Go/No‐Go task (5 stimuli – 2 targets) was positively correlated with activation in the STG, as well as the insula lobe (see Table 7 and Table SM3 in SM).

For both modalities, there was no association between age, socio‐economic status, and BRIEF scores and brain activations related to response inhibition.

## 4 Discussion

This exploratory study investigated the adaptation of the gradCPT to typically developing adolescents and focused on behavioral performance and brain activity patterns across two modalities (*face* and *scene*). Our sample encountered performance challenges, as target detection accuracy was lower than adults. Overall, we demonstrate that adolescents performed better on the *scene* gradCPT. Despite poor behavioral performance, we highlight a bilateral fronto‐parieto‐occipital brain activation during response inhibition, regardless of the type of modality. Furthermore, in the *face* gradCPT, we observed a mainly left‐lateralized pattern in fronto‐cingulo‐cerebellar areas, while in the *scene* gradCPT, an extended bilateral fronto‐temporo‐parieto‐occipital activation was found. Finally, we associated brain activity and behavioral attentional responses.

Our behavioral results showed that participants exhibited a better performance in the *scene* compared to the *face* gradCPT, as evidenced by higher *d’*and lower RT_CV_. The higher *d’* values suggest that participants were more sensitive and demonstrated better discrimination processes during the *scene* gradCPT. We could argue it is because the two stimuli, cities and mountains, are more discernible than male and female faces. This suggests that the bottom‐up attention and the ability to differentiate between stimuli are increased in the *scene* gradCPT, which aligns with other findings in adolescents (Fernandez et al. 2024). Regarding the RT_CV_, lower mean RT variability observed in the *scene* gradCPT reflected better SA performance (Manly et al. 2000; O'Halloran et al. 2018). As suggested by Esterman et al. (2013), low variability could indicate states when motor response settings are optimized to balance speed and accuracy. Therefore, participants were more accurate and able to better adjust their motor responses for cities and mountains stimuli during the *scene* gradCPT. Furthermore, RT_CV_ might also reflect modality complexity, with higher response variability indicating a more cognitively challenging task, as in the *face* modality, where stimuli shared a high degree of similarity.

Accuracy analyses revealed that participants responded precisely to NT stimuli yet faced difficulties to inhibiting responses to T stimuli. In the current study, percentages of correct responses to T stimuli were much lower (*face*: 22.1%; *scene*: 28.1%) than the ones reported in the literature in adult populations using the same paradigm (74%–80%) (Rosenberg et al. 2013; Esterman et al. 2013). This discrepancy may reflect a less mature inhibition system and increased impulsive reactions in adolescence (Fortenbaugh et al. 2015; Halperin et al. 1991), further supported by shorter mean reaction times in our cohort (*face*: 569 ms; *scene*: 577 ms) compared to adults (906 ms) (Rosenberg et al. 2013). Given these performance challenges, caution is needed in interpreting neural activations as reflecting successful sustained attention. However, the preserved NT accuracy (∼87% *face*, ∼94% *scene*) supports that participants did not develop a habitual response strategy (i.e., responding to all stimuli regardless of condition). If participants had responded indiscriminately, NT accuracy would have been similarly low.

RT_CV_ analyses revealed one significant predictor of behavioral performance after FDR correction: the number of errors in the Go/No‐Go task (2 stimuli, 1 target). Conners 3‐SR inattention scores showed a trend‐level association (p (FDR) = 0.157), which should be interpreted with caution. Paradoxically, less impulsive participants (i.e., making fewer errors in the Go/No‐Go task) showed more variability in RT responses and lower gradCPT performance. This can reflect a strategic trade‐off between accuracy and speed: relatively less impulsive participants might be more accurate by responding slower, hence reducing error rate, but increasing RTs variability (Manly et al. 2000; O'Halloran et al. 2018). Instead, more impulsive participants tend to respond faster, increasing error rate but showing more consistent RTs. These findings may appear unintuitive with expectations and call for further explorations. At a trend level, inattentive participants showed higher RT_CV_ (i.e., increased responses variability, lower performance), which is consistent with previous findings linking RT variability to inattention in ADHD individuals (Adams et al. 2011). Taken together, these findings suggest that the number of errors in the Go/No‐Go task is a significant predictor of adolescents’ behavioral performance on the gradCPT paradigm, with Conners inattention scores showing a suggestive trend that requires further investigation.

Given the behavioral performance levels observed, the following activations should be interpreted as showing neural engagement related to inhibition and task effort within a sustained attention context. When investigating the main effect of response inhibition regardless of modality (T > NT), our findings revealed a bilateral fronto‐parieto‐occipito‐thalamic pattern of brain activity, consistent with the SA network described in adults (Langner and Eickhoff 2012) and in young adolescents (Morandini et al. 2020). Strong frontal activity was observed, particularly in the ACC, involved in conflict monitoring, errors detection, and response adjustments (Bediou et al. 2012; Ridderinkhof et al. 2004), and is known to work closely with the anterior insula to detect relevant stimuli and facilitate the processing of task‐related information (Menon and D'Esposito 2022). Parietal regions, such as the SPL and the precuneus, are known to allow top‐down (voluntary) control of attention and attention shifting (Koenigs et al. 2009; Yantis et al. 2002; Yantis and Serences 2003), while the IPS contributes to attentional readjustments in case of attentional drifts, redirection toward the task, and efficient maintenance of SA (Langner and Eickhoff 2012; Posner and Petersen 1990; Corbetta et al. 2008; Malhotra et al. 2009). Further, occipital areas, including the fusiform gyrus, support high‐level visual processes such as stimulus processing and identification (Weiner and Zilles 2016). Moreover, bilateral activation of the posterior cerebellum also corroborates previous studies showing its involvement in cognitive attention processes and SA performance (Langner and Eickhoff 2012; Buckner 2013; Michael et al. 2009; Stoodley 2012). Finally, we observed thalamic activity, which according to previous findings plays a role in maintaining arousal during the task (Langner and Eickhoff 2012). The same study also suggests that the thalamus may contribute to supplementary or compensatory attentional effort (Langner and Eickhoff 2012). These activations, however, cannot be clearly interpreted as indicating successful sustained attention network engagement due to the poor behavioral performance. While not proof of sustained attention network engagement in adolescents, these activations are consistent with it.

When analyzing each of the modalities, the main effect of response inhibition (T > NT) in the *face* gradCPT showed a mainly left‐lateralized pattern of activation in fronto‐cingulo‐cerebellum regions. In contrast, an extended bilateral pattern of fronto‐temporo‐parieto‐occipital activity was recruited during the *scene* gradCPT, with additional brain regions such as the ACC, the anterior insula, the cerebellum, and the thalamus. As previously described, the ACC and the anterior insula are key brain regions for SA and were already associated with good performance in both healthy and ADHD adolescents during a Go/No‐Go task (O'Halloran et al. 2018). These differences may come from differences in perceptual demands, as face discrimination (male vs. female) may engage more focused processing in face‐selective regions, whereas scene discrimination (city vs. mountain) requires distributed spatial and semantic analysis involving larger networks. Moreover, such broader brain activation observed only during the *scene* gradCPT aligns with our previous premise: better task performance might be explained by greater stimulus distinction, with city and mountain stimuli being notably more distinguishable than those involving male and female faces. Finally, the difference in cluster sizes between the *scene* and *face* modalities is consistent with the significant behavioral performance difference (*scene d*' = 1.12 vs. *face d*' = 0.44). Better behavioral performance in the *scene* modality is linked with more spatially coherent and consistent BOLD responses at the group level, whereas the high inter‐individual variability in *face* task performance results in weaker and more spatially constrained group‐level activation.

We also observed positive correlations between brain activity and behavioral attentional performance (for both *d’* scores and RT_CV_), as well as Conners 3‐SR inattention scores and TAP scores in Go/No‐Go tasks. Regarding the gradCPT performance, adolescents with higher *d’* scores displayed stronger activation in the left ACC during response inhibition in the *scene* gradCPT, specifically. This aligns with the study of O'Halloran et al. (2018), who reported that increased bilateral ACC activity was linked to better performance in SA tasks. As discussed previously, left brain region activation might reflect the challenging nature and high cognitive demands of such tasks. Furthermore, adolescents with higher RT_CV_ (i.e., higher variability in RT responses and poor performance) showed increased brain activity in frontal, parietal, and temporal areas in the *face* gradCPT. This is consistent with prior studies that linked increased behavioral variability in inhibition tasks with increased activity in fronto‐parietal areas (Bellgrove et al. 2004; Simmonds et al. 2007). In this study, regarding the *scene* gradCPT, higher RT variability was associated with higher activity in the left posterior cerebellum. While a previous Go/No‐Go study reported a similar association in the right posterior cerebellum (Simmonds et al. 2007), our findings are the first to demonstrate a correlation between RT variability and the left posterior cerebellum, which needs confirmation and further investigations.

Our investigations of covariate effects on SA cerebral activation revealed that adolescents with higher inattention scores displayed increased activity in frontal, temporal, parietal, and occipital brain regions and the right posterior insula during the *face* gradCPT. In the *scene* gradCPT, we found an association between inattention scores and brain activity in parietal and occipital regions, as well as the left posterior cerebellum. Thus, more inattentive adolescents might use compensatory mechanisms to perform the task similarly to the less inattentive ones, as shown by increased activity in brain regions typically involved in SA processes.

Correspondingly, we found that brain activity during both modalities was positively associated with the performance in Go/No‐go tasks from the TAP. We observed a stronger brain activity among adolescents who made more errors, which can further support the above assumption of them needing to compensate for greater impulsivity and possibly deficits in inhibitory control. Furthermore, error‐related brain activity following failures to inhibit a response in Go/No‐Go tasks engages regions that partially overlap with those involved in response inhibition (Menon et al. 2001). With this, the following hypothesis could be proposed: adolescents, due to impulsive reactions, struggled to inhibit their dominant responses and were conscious of the errors they made. Consequently, brain regions related to error processing were more prominently engaged in response to these errors. This interpretation is consistent with two complementary frameworks. First, the ACC has been proposed to integrate expected benefits and effort costs to allocate cognitive control, and its activation may reflect effortful control demands (Shenhav et al. 2013). Second, electrophysiological evidence links ACC activity and frontal midline theta oscillations to increased cognitive effort required to sustain performance over time (Umemoto et al. 2019). These frameworks support the interpretation that ACC activations in our study may partly reflect effortful compensatory engagement in response to repeated inhibitory failures.

Given the performance issues that were encountered, our findings should be interpreted carefully. Definitive conclusions regarding successful sustained attention network engagement in adolescents cannot be made due to the low target detection accuracy. Nevertheless, our findings provide some insights in identifying specific implementation challenges that need to be addressed for the gradCPT paradigm. Rather than a failed attempt, this exploratory study provides a methodological roadmap and opens opportunities for future research to refine the gradCPT for adolescent populations.

### 4.1 Limitations

Our results demonstrated lower accuracy across both modalities, compared to adult literature, suggesting adolescents’ difficulties with response inhibition, as also concluded by Fernandez et al. (2024). Thus, all target trials had to be included in the whole‐brain analyses, rather than only correct ones. One potential reason for the poor task performance is the task's complexity. Nevertheless, error rates did not suggest random responding or lack of engagement, and importantly, the observed brain activity patterns are consistent with prior findings related to SA. Furthermore, while performance in the *face* gradCPT was lower than expected, *d’* scores in the *scene* gradCPT aligned with previous studies in adults (M  =  1.12) (Rosenberg et al. 2013). Furthermore, compared to factorial go/no‐go designs (Hwang et al. 2019), the structure of the gradCPT does not allow a clear dissociation of SA and response inhibition. In this study, the T > NT contrast should therefore be understood as capturing both processes together (Hwang et al. 2019). A detailed explanation of limitations can be found in S2 in the SM.

In conclusion, this exploratory study provides preliminary observations of the neural substrates of SA and inhibition processes in typically developing adolescents using the gradCPT paradigm. More specifically, and consistent with previous studies in adults, we observed brain activity related to SA in bilateral fronto‐parieto‐occipital brain regions. In addition, these results may help in our understanding of attentional difficulties and call for future research to explore preventive and therapeutic approaches.

## Author Contributions

**Jade Awada**: investigation, writing – original draft, methodology, validation, visualization, formal analysis, data curation. **Natalia Fernandez**: investigation, writing – original draft, methodology, validation, conceptualization. **Vanessa Siffredi**: conceptualization, writing – review and editing. **Jenifer Miehlbradt**: writing – review and editing. **Cristina Borradori‐Tolsa**: writing – review and editing. **Maria Chiara Liverani**: writing – review and editing. **Russia Ha‐Vinh Leuchter**: writing – review and editing, supervision, funding acquisition, project administration, resources.

## Funding

This work was supported by the Swiss National Science Foundation (SNF; fund number: 320030B_182832), and the Gertrude Von Meissner Foundation (fund number: F02‐12587).

## Conflicts of Interest

The authors declare no conflicts of interest.

## Supporting information




**Supplementary Materials**: brb371516‐sup‐0001‐SuppMat.docx

## Data Availability

The data that support the findings of this study are available from the corresponding author upon reasonable request.
