# Supplementary material for: Brain Activity During the Gradual‐Onset Continuous Performance Task in Adolescents: An Exploratory fMRI Study
Source: Brain Behav. 2026 Jun 4;16(6):e71516. doi: 10.1002/brb3.71516 (PMC13580856; doi:10.1002/brb3.71516)
Supplement: Supplementary file 1 [file BRB3-16-e71516-s001.docx]

**Supplemental Materials**

**Brain Activity During the Gradual-Onset Continuous Performance Task in Adolescents: An Exploratory fMRI**

J. Awada^1,2^*, NB. Fernandez^1^*, V. Siffredi^1,3,4^, J. Miehlbradt^1^, C. Borradori‑Tolsa^1^, MC. Liverani^1,5^ & R. Ha-Vinh Leuchter^1^

^1^ Division of Development and Growth, Department of Paediatrics, Gynaecology and Obstetrics, Geneva University Hospitals, Geneva, Switzerland

^2^ Neuroimaging and translational psychiatry lab, Department of psychiatry, University of Geneva, Switzerland

^3^ Institute of Bioengineering, Center for Neuroprosthetics, Ecole Polytechnique Fédérale de Lausanne, Switzerland

^4^ Department of Radiology and Medical Informatics, Faculty of Medicine, University of Geneva, Switzerland

^5^ SensoriMotor, Affective and Social Development Laboratory, Faculty of Psychology and Educational Sciences, University of Geneva, Geneva, Switzerland

*Shared authorship

Table of contents

[S1. Rationale for stimulus selection	3](#_Toc228297178)

[S2. Full details of methods	4](#_Toc228297179)

[Data collection	4](#_Toc228297180)

[Executive and attentional behavioral measures	4](#_Toc228297181)

[Conners Rating scale third edition (Conners 3-SR).	4](#_Toc228297182)

[Behavior Rating Inventory of Executive Functioning (BRIEF)	5](#_Toc228297183)

[Test of Attentional Performance (TAP)	5](#_Toc228297184)

[Experimental fMRI paradigm: Gradual-Onset Continuous Performance task	6](#_Toc228297185)

[Behavioral data analyses	8](#_Toc228297186)

[fMRI analyses	9](#_Toc228297187)

[Functional Magnetic Resonance Imaging (MRI) acquisition	9](#_Toc228297188)

[MRI data preprocessing	9](#_Toc228297189)

[fMRI analysis	10](#_Toc228297190)

[Participants Characteristics	11](#_Toc228297191)

[S3. Analysis of modality order effect	13](#_Toc228297192)

[S4. Extended Discussion of Limitations	14](#_Toc228297193)

[S5. Additional analysis: Influence of sex and age on neural activation	16](#_Toc228297194)

[Bibliography	17](#_Toc228297195)

[TABLES	18](#_Toc228297196)

# S1. Rationale for stimulus selection

Face and scene processing engage partially distinct neural pathways and differ in their perceptual and cognitive demands (1,2). We hypothesized that processing differences might interact with developmental factors to produce differential sustained attention performance.

Both face and scene modalities were selected, because they represent ecologically valid stimuli that are frequently encountered in everyday life. While scene stimuli reflect the constant visual environments experienced in daily life, face stimuli are common in social communication and interactions and are already frequently used in attention studies. Thus, adolescents may exhibit different performance or activation patterns based on stimulus category, as face and scene processing involve different neural pathways (ventral vs. dorsal streams). Furthermore, to our knowledge, the face modality has never been used in fMRI studies involving adults with the gradCPT, even though the scene modality has been used previously. We aimed to fill this gap by incorporating the face modality and investigating whether this socially relevant category could elicit sustained attention patterns.

# S2. Full details of methods

## Data collection

Twenty-nine adolescents aged 11-18 years old (M = 15 years, SD = 2) recruited from the community between January 2021 and August 2022 took part in the study. All participants were typically developing and attended mainstream schools. Participants with an intelligence quotient below 70, any sensory or physical impairments (e.g., blindness, hearing loss, cerebral palsy), or an insufficient understanding of French or English were excluded. General intellectual functioning was derived from the General Ability Index (GAI) of the Wechsler Intelligence Scale for Children - 5th Edition (WISC-V)(3). The GAI has a mean of 100 and a standard deviation of 15. The Largo score is an estimate of socio-economic status based on maternal education and paternal occupation (4). Higher socio-economic scores indicate lower socio-economic status levels.

The current study was approved by the Swiss Ethics Committee on research involving human beings (ID: 2015-00175). All participants and their principal caregiver signed an informed consent form approved by the ethics committee at the University Hospital of Geneva. Each participant was rewarded with a gift voucher worth 50 Swiss francs for their participation.

## Executive and attentional behavioral measures

### Conners Rating scale third edition (Conners 3-SR).

The *Conners self-report questionnaire* assesses adolescents’ attentional abilities in their daily lives and allows detection of symptoms related to attention disorders, with or without hyperactivity. It consists of 97 questions, and categorizing the items results in two subscales: the inattention scale and the hyperactivity/impulsivity scale. Results are presented as T-scores.

### Behavior Rating Inventory of Executive Functioning (BRIEF)

Parents completed the *BRIEF* questionnaire, which measures executive functions within home environment for children and adolescents aged 5-18 years (5). The BRIEF questionnaire is commonly used in clinical assessments and consists of 8 executive functioning scales: emotional control, inhibition, flexibility, initiation, material organization, working memory, planning/organization, and control. These scales can be categorized into two standardized subscales: 1) the *Behavioral Regulation Index (BRI)*, which combines inhibition, flexibility, and emotional control scales; and 2) the *Metacognition Index (MI),* which combines initiation, material organization, working memory, planning/organization, and control scales. The *Global Executive Composite index (GEC)* is derived from the sum of scores from all 8 scales. We used T-scores of these three indexes (BRI, MI, and GEC) to assess the executive abilities of our typically developing adolescents.

### Test of Attentional Performance (TAP)

To assess attentional abilities, computerized tasks of the Test of Attentional Performance (TAP) (6) were administered with oral instructions provided by the experimenter. To assess inhibitory control, we administered two *Go/No-Go* paradigms: one with 1 target against 3 stimuli and another one with 2 targets against 5 stimuli. We then analyzed the number of errors, which are indicative of impulsivity and control deficits (6).

## Experimental fMRI paradigm: Gradual-Onset Continuous Performance task

Stimuli presentation and response recording through MRI-compatible response buttons were controlled with a Psychophysics Toolbox extension (<https://psychtoolbox.org>) implemented in Matlab 2019b (Mathworks Inc., Natick, MA, USA).

Two distinct versions of the gradual-onset continuous performance task (gradCPT) were used in the current study, a paradigm that has been previously validated and fully described by Rosenberg and colleagues (1,2). Each version represents distinct modalities, the *face* and *scene* modalities: in the *face* modality, female faces were considered as target trials and male faces as non-target trials, while in the *scene* modality, mountain images were considered as target trials and city images as non-target trials (see *Figure 1*) (7).

Both versions of the gradCPT task had a duration of six minutes each and visual stimuli gradually transitioned between the target and non-target stimuli at a constant rate, in accordance with the original gradCPT paradigm (Esterman et al., 2013). Each trial had an effective duration of approximately 600ms, as derived from the acquisition parameters (500 task volumes × TR 720ms / 600 trials). In each modality, “visual stimuli were randomly presented” and target trials were presented on 10% of the trials (60 trials) while non-target were presented 90% of the time (540 trials) (7), so that there was no repetition of stimuli in two subsequent trials. Participants were asked to respond as fast and as accurately as possible to non-target stimuli (i.e., male faces or city) by pressing the response button and to withhold response to target stimuli (i.e., female faces or mountains) (1).

In the *scene* modality, there are 10 distinct images of mountains (targets) and 10 distinct images of cities (non-targets). Throughout the session, each city image appeared 54 times and each mountain image appeared 6 times, with 60 target trials and 540 non-target trials. Images were randomly presented with the constraint that identical scenes are not repeated on consecutive trials. The *face* modality follows the same structure, with 10 images of male faces and 10 images of female faces. On both modalities, stimuli were presented via MR-compatible goggles (NordicNeuroLab Visual System HD, resolution 1920×1080 at 60Hz refresh rate). Visual presentation parameters were: screen dimensions: 509 × 286 mm (approximately 23 inches diagonal), viewing distance: approximately 1350 mm, stimulus size: 256 pixels diameter (67.8 mm), visual angle: approximately 2.9° of visual angle. Participants were given thorough verbal instructions about the task outside the scanner room before they entered the scanner. They watched a brief demonstration video that showed examples of target and non-target stimuli and the gradual transition mechanism for both modalities (face and scene conditions) and also practiced a few trials on a mock scanner to ensure they had properly understood the task. All participants performed the task in the scanner with the right hand. The order of the two modalities was counterbalanced across participants (n = 13 completed the *face* modality first; n = 13 completed the *scene* modality first). A resting-state fMRI scan was administered between the two runs, during which participants were instructed to rest with their eyes closed. For a full description on trial assessment and response validation, please refer to Rosenberg et al. (2013).

## Behavioral data analyses

Sustained attentional performance was assessed by the percentage of correct answers (accuracy, AC), sensitivity (*d’*), and the reaction times coefficient of variation (RT_CV_). All statistical analyses were performed using RStudio version 4.2.1 (8) and Statistica software version 14.0.0 (9).

For each participant and each modality, we calculated the *accuracy* (AC) for both the non-target and target trials separately, which were analysed using a Mann-Whitney test with modality (*face* and *scene*) and stimuli (non-target and target) as within-subject levels.

*d’* score is a measure of performance and considers the proportion of correct non-target (*HIT rate*) and the proportion of incorrect target (*FALSE ALARM rate*). It was calculated for each participant and each modality separately using the following formula: *d’= z(HIT rate) - z(FALSE ALARM rate)*(1,2,10). We compared sustained attention performances between *face* and *scene* modalities with *d’* scores using a simple t-test.

Previous studies have found that reaction times (RT) variability is a significant indicator of attentional state and a unique component of the performance of sustained attention (1,10). RT_CV_ was calculated, for correct non-target stimuli for each participant and for each modality independently, by dividing the standard deviation of reaction times (RTs) by the mean RTs (10–12). We compared RT_CV_ across modalities with simple t-tests.

Additionally, we examined the influence of age, socio-economic status (measured by the Largo scale), executive functions (evaluated with the BRIEF) and attentional abilities (evaluated with the Conners 3-SR and TAP) on sustained attentional performance. To account for the within-subject repeated measures structure (i.e., face and scene modalities per participant), we conducted a linear mixed effects model (fitlme, MATLAB Statistics Toolbox, version R2022a) with RTCV as the dependent variable, Task (face vs. scene), age, socio-economic status, IAG, BRIEF subscales (GEC, MI, BRI), Conners 3-SR (inattention and hyperactivity) and TAP scores (divided attention, Go/No-Go simple and hard, working memory) as fixed effects, and Subject as a random intercept. A separate analysis with d' as dependent variable was also conducted. P-values were corrected for multiple comparisons using the Benjamini-Hochberg FDR procedure.

## fMRI analyses

### Functional Magnetic Resonance Imaging (MRI) acquisition

MRI data were acquired at the Campus Biotech (Geneva, Switzerland) using a Siemens 3T Magnetom Prisma scanner and a standard 64‑channel head coil. Structural T1-weighted magnetization-prepared rapid gradient-echo (MP-RAGE) sequences were acquired using the following parameters: voxel size = 0.9 × 0.9 × 0.9 mm, TR/TE = 2300/ 2.32 ms, flip angle = 8°, and FOV = 240 x 240 mm. Functional T2*-weighted images were acquired using multi-slice gradient-echo-planar imaging (EPI) sequences and the following parameters: voxel size = 2 x 2 x 2 mm, TR/TE = 720/33ms, flip angle = 52°, and FOV = 208 x 208 mm. In addition, a fieldmap (TR/TE_1_/TE_2_ = 627/5.19/7.65 ms, flip angle = 60°, voxel size = 2 x 2 x 2 mm, and FOV = 208 x 208 mm) was obtained for each participant.

### MRI data preprocessing

Statistical Parametric Mapping software (SPM12; Wellcome Department of Imaging Neuroscience, UCL, UK) implemented in MATLAB R2019a (The MathWorks, Inc., Natick, Massachusetts, United States) was used for the preprocessing and analysis of MRI images.

Following an in-house preprocessing pipeline (13,14), fMRI images were spatially realigned and unwarped to correct for motion artifacts and geometric distortions (15) for each participant. Functional images were then co-registered with the structural images in the individual’s own brain space. Structural images were segmented using SPM12 segmentation routines to produce separate tissue probability maps (TPMs) including grey matter, white matter, and cerebrospinal fluid (16), and a study-specific template was created using Diffeomorphic Anatomical Registration using Exponential Lie algebra (DARTEL) (17). Eventually, functional images were normalized to the DARTEL template and smoothed with a 6 mm full width at half maximum (FWHM) isotropic Gaussian kernel. Temporal high-pass filtering was applied using SPM's default filter with a cutoff of 128 seconds to remove low-frequency drifts. As previously applied by researchers in our lab (13,14), head motion was evaluated using Framewise Displacement (FD)(18), computed for each modality (*face* or *scene*) separately and used as an exclusion criterion. Only one participant was excluded from the current study as more than 20% of his frames showed excessive motion (frames with FD greater than 0.5mm, including one frame before and two after those).

### fMRI analysis

Individual first level analyses were performed with a General Linear Model (GLM). The model included the two modalities with target (T) and non-target (NT) conditions, and six realignment parameters (head motions) entered as non-interest covariates. Trial onsets were aligned with the beginning of image changes, lasting for 0.57 seconds, and adjusted using the canonical hemodynamic response function (cHRF). For each subject, first-level T-contrasts were determined by comparing the two experimental conditions (T > NT) and the two experimental modalities (*face* > *scene* or *scene* > *face*).

Resulting first-level statistical parametric maps were entered into a second-level (whole-brain) flexible factorial model that included modality (*face* and *scene*), stimuli (T and NT), and subject as separate factors. First, the main effect of task was measured by comparing the two modalities (*face* > *scene* or *scene* > *face*). Second, the main effect of response inhibition was measured by comparing the target to non-target conditions (T > NT). Then, the effect of response inhibition on modality was measured by contrasting modalities and stimuli (*face* (T > NT) > *scene* (T > NT) or *scene* (T > NT) > *face* (T > NT)). The direction of the interaction was confirmed by applying the effect of response inhibition for a specific task (*face* (T > NT) or *scene* (T > NT) respectively) as an inclusive mask to restrict the direction of the contrast.

Moreover, using separated univariate linear regression models, we investigated how cerebral activity during response inhibition varied as a function of covariates of interest (i.e., *d’* scores and RT_CV_, age at testing, socio economic status, as well as BRIEF, Conners 3-SR, and TAP scores). We extracted individual beta values associated with significant peak activations from the univariate linear regression analyses, by employing spheres (27 voxels) centered on the main peak coordinates identified by covariates SPM analyses. Subsequent correlation analyses between beta values and covariates of interest were performed in R Studio.

For second-level whole-brain analyses, we reported p-values with a statistical threshold of p_unc_ < 0.001 uncorrected for multiple comparisons, with a minimum size of 50 voxels (19). All findings are presented in MNI space.

## Participants Characteristics

One participant was excluded from final analyses as he did not finish the scanner session, one participant was excluded for excessive movement, and one for lack of withholding response during the task. The final sample included 26 participants, and was balanced between male (n=13, 50%), and female (n=13, 50%). Participants presented a mean general ability index of 110.36 (SD = 11.11) and a mean socio-economic status (Largo score) of 3.00 (SD = 1.57).

# S3. Analysis of modality order effect

To examine modality order effects, independent samples t-tests were conducted on d' scores comparing participants who completed the *face* modality first (n = 13) with those who completed the *scene* modality first (n = 13), separately for each modality. A Task × Order interaction was also tested by computing t-tests on within-subject Scene–Face d' differences between the two groups.

Results revealed no significant order effect on *face* modality performance (t(24) = 0.576, p = .570), no significant order effect on *scene* modality performance (t(24) = 0.836, p = .412), and no significant Task × Order interaction (t(24) = 0.355, p = .725). The main effect of Task (*face* vs. *scene*) remained highly significant (t(25) = −5.115, p < .001), consistent with our original findings. These results confirm that task order did not confound the behavioral results.

# S4. Extended Discussion of Limitations

In contrast to Rosenberg et al. (2013) findings in adults, our study encountered challenges with adolescent participants. The percentage of correct target trials was lower for both modalities (scene and face), meaning that adolescents struggled in inhibiting responses. Therefore, rather than selecting only the correct target trials for whole-brain activation investigations, we included all target trials. As target detection accuracy was lower than in the adult population, this requires cautious interpretation of neural activation patterns.

One potential reason for poor task performance may be related to the complexity of the task itself: as previously mentioned, adolescents are more likely to give more impulsive responses and might have difficulties in correctly withholding responses to target stimuli specifically. However, we do not believe that participants were disinterested in the gradCPT, or that their responses were random. In a case of random responses, the error rate in the nontarget condition (omission errors) would have been close to 50%, which is greater than ours, and a lack of interest in the task would not have induced brain activity associated with response inhibition. Moreover, our findings on neural substrates of sustained attention and inhibition are in line with those found in previous studies. Furthermore, our behavioral performance scores (*d’* specifically) during the *scene* gradCPT are close to those found in previous studies in adults (M = 1.12)(1). For the *face* gradCPT specifically, there are no comparable studies available, but we recognize that behavioral performance scores were lower than expected and task-induced brain activity seemed to be weaker compared to the *scene* gradCPT. There are limited comparative data of gradCPT studies in adolescents. Crucially, d' ≈ 2.0 was reported by Fortenbaugh et al. (2015) in children (10–12 years), which is significantly higher than our adolescent sample (d' = 1.12, scene).

The findings should therefore be interpreted as exploratory and preliminary. Activations are consistent with sustained attention regions, but given performance levels, we cannot conclude that adolescents successfully engaged these networks. Future research is thus needed to validate the gradCPT in adolescents.

A further limitation is that pubertal status was not assessed in the present study. Our sample comprised a wide age range (11–18 years, M = 15, SD = 2), and participants were at very different stages of pubertal development. Although age was included as a covariate in our analyses and did not emerge as a significant predictor of behavioral or neural outcomes, pubertal maturation is only partially correlated with chronological age. Two adolescents of the same age may differ in their pubertal stage, and these differences can influence the maturation of prefrontal and fronto-parietal networks. Future research should consider including measures of pubertal development to better account for developmental factors that may influence sustained attention performance in adolescents.

# S5. Additional analysis: Influence of sex and age on neural activation

To address potential sex-related differences in neural activation patterns during the gradCPT task, we conducted additional second-level analyses including sex and age as covariates. As males and females may engage partially distinct neural networks during cognitive control tasks, we included sex as a nuisance covariate at the second level to account for potential confounds in regional activation patterns.

We performed a two-sample t-test comparing males (n = 13) and females (n = 13) on the Target>NonTarget (T>NT) contrast, with age included as a covariate, to investigate possible sex differences in neural mechanisms during face and scene processing under conditions requiring response inhibition.

We focused on the interaction between group (males vs. females) × stimulus type (T>NT). To confirm the direction of any potential interaction, we applied the within-group effect for each sex (males T>NT or females T>NT, respectively) as an inclusive mask using a threshold of p = 0.001 uncorrected.

Using a threshold of p < 0.001 (uncorrected) and a cluster extent of k = 50 voxels, no suprathreshold clusters were identified. This indicates that the T>NT contrast in our sample was not significantly impacted by either sex or age. These results indicate that the T>NT effect observed in our sample was not modulated by sex when controlling for age and thus show comparable neural mechanisms underlying response inhibition between males and females in our adolescent sample.

# Bibliography

1.	Rosenberg M, Noonan S, DeGutis J, Esterman M. Sustaining visual attention in the face of distraction: a novel gradual-onset continuous performance task. Atten Percept Psychophys. avr 2013;75(3):426‑39.

2.	Rosenberg MD, Finn ES, Scheinost D, Papademetris X, Shen X, Constable RT, et al. A neuromarker of sustained attention from whole-brain functional connectivity. Nat Neurosci. janv 2016;19(1):165‑71.

3.	Wechsler D. Wechsler Intelligence Scale for Children. 5th éd. MN: PsychCorp; 2014. (Bloomington).

4.	Largo R, Pfister D, Molinari L, Kundu S, Lipp A. Significance of prenatal, perinatal and postnatal factors in the development of AGA preterm infants at five to seven years. Dev Med Child Neurol. 1989;31(4):440‑56.

5.	Gioia, Isquit, Guy, Kenworthy, Roy, Fournet, et al. BRIEF : Inventaire d’Évaluation Comportementale des Fonctions Exécutives. Editions Hogrefe France. 2014.

6.	Zimmermann, Fimm. Test of Attentional Performance (TAP) - Manual. Würselen. Germany: Psytest.; 2002.

7.	Fernandez NB, Siffredi V, Awada J, Miehlbradt J, Borradori-Tolsa C, Liverani MC, et al. Sustained attention processes in very preterm adolescents and their relationship to socio-emotional competence. medRxiv. 1 janv 2024;2024.09.30.24314596.

8.	R Core Team. R: A language and environment for statistical computing. 2022; Disponible sur: https://www.R-project.org/

9.	TIBCO Statistica [Internet]. Palo Alto, CA, USA: TIBCO Software Inc; 2020. Disponible sur: https://docs.tibco.com/

10.	Rosenberg MD, Finn ES, Constable RT, Chun MM. Predicting moment-to-moment attentional state. NeuroImage. juill 2015;114:249‑56.

11.	Fortenbaugh FC, Rothlein D, McGlinchey R, DeGutis J, Esterman M. Tracking behavioral and neural fluctuations during sustained attention: A robust replication and extension. Neuroimage. 1 mai 2018;171:148‑64.

12.	Urbain C, Sato J, Hammill C, Duerden EG, Taylor MJ. Converging function, structure, and behavioural features of emotion regulation in very preterm children. Human Brain Mapping. août 2019;40(11):3385‑97.

13.	Freitas LGA, Liverani MC, Siffredi V, Schnider A, Borradori Tolsa C, Ha-Vinh Leuchter R, et al. Altered orbitofrontal activation in preterm-born young adolescents during performance of a reality filtering task. NeuroImage: Clinical. 2021;30:102668.

14.	Liverani MC, Freitas LGA, Siffredi V, Mikneviciute G, Martuzzi R, Meskaldij D, et al. Get real: Orbitofrontal cortex mediates the ability to sense reality in early adolescents. Brain Behav [Internet]. avr 2020 [cité 24 mars 2023];10(4). Disponible sur: https://onlinelibrary.wiley.com/doi/10.1002/brb3.1552

15.	Hutton C, Bork A, Josephs O, Deichmann R, Ashburner J, Turner R. Image Distortion Correction in fMRI: A Quantitative Evaluation. NeuroImage. mai 2002;16(1):217‑40.

16.	Ashburner J, Friston KJ. Unified segmentation. NeuroImage. juill 2005;26(3):839‑51.

17.	Ashburner J. A fast diffeomorphic image registration algorithm. NeuroImage. oct 2007;38(1):95‑113.

18.	Power JD, Barnes KA, Snyder AZ, Schlaggar BL, Petersen SE. Spurious but systematic correlations in functional connectivity MRI networks arise from subject motion. NeuroImage. févr 2012;59(3):2142‑54.

19.	Lieberman MD, Cunningham WA. Type I and Type II error concerns in fMRI research: re-balancing the scale. Soc Cogn Affect Neurosci. déc 2009;4(4):423‑8.

20.	Fortenbaugh FC, DeGutis J, Germine L, Wilmer JB, Grosso M, Russo K, et al. Sustained Attention Across the Life Span in a Sample of 10,000: Dissociating Ability and Strategy. Psychol Sci. sept 2015;26(9):1497‑510.

# TABLES

**Table SM1: Pearson’s correlations between individual Beta values and d’ and RT_CV_ scores during the GradCPT tasks. (*) significant p-value.**

**Table SM2: Pearson’s correlations between individual Beta values and Conners’ inattention scores during the GradCPT tasks. (*) significant p-value.**

**Table SM3: Pearson’s correlations between individual Beta values and TAP scores during the GradCPT tasks.**

**Table SM1: Pearson’s correlations between individual Beta values and d’ and RT_CV_ scores during the GradCPT tasks. (*) significant p-value.**

| **Cerebral regions** | |  |  |  |
| --- | --- | --- | --- | --- |
|  |  | **Lat.** | **Pearson’s correlation coefficient (r)** | **p-value** |
| Face task | **Association with RT_CV_** |  |  |  |
| *Frontal* | Superior frontal gyrus | L | .589 | .0002* |
|  | Superior frontal gyrus | R | .709 | <.0001* |
|  | Posterior-medial frontal – dACC/SMA | R | .633 | .001* |
| *Temporal* | Middle temporal gyrus | L | .655 | .0004* |
|  | Superior temporal gyrus | L | .697 | .0001* |
| *Parietal* | Angular gyrus | L | .673 | .0002* |
|  | Angular gyrus | R | .710 | <.0001* |
|  |  |  |  |  |
| Scene task | **Association d’ scores** |  |  |  |
| *Other* | Anterior cingulate cortex | L | .597 | .002* |
|  | **Association with RT_CV_** |  |  |  |
| *Other* | Cerebellum (VI) | L | .396 | .050* |

**Table SM2: Pearson’s correlations between individual Beta values and Conners’ inattention scores during the GradCPT tasks. (*) significant p-value.**

| **Cerebral regions** | |  |  |  |
| --- | --- | --- | --- | --- |
|  |  | **Lat.** | **Pearson’s correlation coefficient (r)** | **p-value** |
| Face task | **Association with Conners’ inattention scores** |  |  |  |
| *Frontal* | Superior precentral gyrus | L | 0.337 | 0.100 |
| *Temporal* | Superior temporal gyrus | L | 0.319 | 0.120 |
| *Parietal* | Superior precuneus | L | 0.444 | 0.026* |
|  | Superior parietal lobule | L | 0.412 | 0.041* |
|  | Superior parietal lobule | R | 0.504 | 0.010* |
| *Occipital* | Calcarine sulcus | L | 0.752 | <.001* |
|  | Calcarine sulcus | R | 0.584 | 0.002* |
|  | Superior cuneus | L | 0.465 | 0.019* |
|  | Superior cuneus | R | 0.551 | 0.004* |
|  | Lingual gyrus | L | 0.494 | 0.012* |
|  | Superior occipital gyrus | L | 0.498 | 0.011* |
|  | Superior occipital gyrus | R | 0.425 | 0.034* |
| *Other* | Insula lobe – posterior insula | R | 0.469 | 0.018* |
|  | Parahippocampal gyrus | L | 0.602 | 0.002* |
|  |  |  |  |  |
| Scene task |  |  |  |  |
| *Parietal* | Precuneus | L | 0.401 | 0.047* |
| *Occipital* | Calcarine sulcus | L | 0.316 | 0.124 |
|  | Cuneus | L | 0.149 | 0.479 |
|  | Cuneus | R | 0.343 | 0.094 |
|  | Inferior lingual gyrus | L | 0.342 | 0.095 |
|  | Inferior lingual gyrus | R | 0.205 | 0.325 |
|  | Superior occipital gyrus | L | 0.286 | 0.162 |
|  | Superior occipital gyrus | R | 0.415 | 0.039* |
| *Other* | Cerebellum (VI) | L | 0.333 | 0.104 |

**Table SM3: Pearson’s correlations between individual Beta values and TAP scores during the GradCPT tasks.**

| **Cerebral regions** | |  |  |  |
| --- | --- | --- | --- | --- |
|  |  | **Lat.** | **Pearson’s correlation coefficient (r)** | **p-value** |
| Face task | **Association with Go/No-go scores (2 stim – 1 target)** |  |  |  |
| *Parietal* | Inferior precuneus | L | 0.655 | <0.001* |
| *Occipital* | Inferior cuneus | L | 0.694 | <0.001* |
| *Subcortical* | Basal ganglia – putamen | L | 0.704 | <0.001* |
|  |  |  |  |  |
|  | **Association with Go/No-go scores (5 stim – 2 targets)** |  |  |  |
| *Frontal* | Superior precentral gyrus | L | 0.661 | <0.001* |
|  |  |  |  |  |
| Scene task | **Association with Go/No-go scores (5 stim – 2 targets)** |  |  |  |
| *Temporal* | Superior temporal gyrus | R | 0.752 | <0.001* |
| *Other* | Insula lobe – posterior insula | R | 0.656 | <0.001* |
